# Extracting Message Inter-Departure Time Distributions from the Human Electroencephalogram

**DOI:** 10.1371/journal.pcbi.1002065

**Published:** 2011-06-02

**Authors:** Bratislav Mišić, Vasily A. Vakorin, Nataša Kovačević, Tomáš Paus, Anthony R. McIntosh

**Affiliations:** 1 Rotman Research Institute, Baycrest Centre, Toronto, Ontario, Canada; 2 Department of Psychology, University of Toronto, Toronto, Ontario, Canada; 3 Cognitive Neuroscience Unit, Montreal Neurological Institute, McGill University, Montreal, Quebec, Canada; University of Cambridge, United Kingdom

## Abstract

The complex connectivity of the cerebral cortex is a topic of much study, yet the link between structure and function is still unclear. The processing capacity and throughput of information at individual brain regions remains an open question and one that could potentially bridge these two aspects of neural organization. The rate at which information is emitted from different nodes in the network and how this output process changes under different external conditions are general questions that are not unique to neuroscience, but are of interest in multiple classes of telecommunication networks. In the present study we show how some of these questions may be addressed using tools from telecommunications research. An important system statistic for modeling and performance evaluation of distributed communication systems is the time between successive departures of units of information at each node in the network. We describe a method to extract and fully characterize the distribution of such inter-departure times from the resting-state electroencephalogram (EEG). We show that inter-departure times are well fitted by the two-parameter Gamma distribution. Moreover, they are not spatially or neurophysiologically trivial and instead are regionally specific and sensitive to the presence of sensory input. In both the eyes-closed and eyes-open conditions, inter-departure time distributions were more dispersed over posterior parietal channels, close to regions which are known to have the most dense structural connectivity. The biggest differences between the two conditions were observed at occipital sites, where inter-departure times were significantly more variable in the eyes-open condition. Together, these results suggest that message departure times are indicative of network traffic and capture a novel facet of neural activity.

## Author Summary

The brain may be thought of as a network of regions that communicate with each other to produce emergent phenomena such as perception and cognition. Many potentially interesting aspects of brain networks, such as how information is emitted at different nodes, also tend to be of interest in various types of telecommunication systems, such as telephony. Thus, network properties that are relevant in the context of brain function may be important for telecommunication networks in general. Here we show how neural activity can be partitioned into units of information and analyzed from the perspective of a telecommunication system. We demonstrate that the inter-departure times of such units of information have very similar probability distributions across subjects and that they are sensitive both to regional variation and cognitive state. The approach we describe can be applied in a wide variety of experimental paradigms to generate novel indices of neural activity and open new avenues for network analysis of the brain.

## Introduction

Recent years have witnessed a remarkable drive to characterize the large-scale structural topology of the brain. The graph model of cortical connectivity – whereby space is discretized and the brain is delineated as a set of regional nodes interconnected by white matter edges – has enabled the application of a whole host of network metrics [1], [2]. The cerebral connectome [3] has been found to possess highly nontrivial properties that do not appear in random networks with comparable connection density and could potentially endow it with a greater capacity to process information. These include small-worldness [4]–[6] and the presence of hubs [7], [8].

However, the functional consequences of this structural foundation are less clear and in general the translation from structure to function has been more difficult to understand. The emergent functional connectome has hitherto been studied by applying similar network analytic measures to graphs extracted from functional data. One approach has been to use these indices as a basis of comparison between networks defined by structural and functional connections. For example, physical links between nodes certainly beget sustained functional interactions and as a result functional brain networks map onto the underlying structural architecture to a great extent [8]–[10]. Another approach has been to study functional networks exclusively and without explicit reference to the underlying structural networks [11], [12].

An important aspect of brain network organization that remains to be investigated is the throughput of information at individual nodes. How does the flux of information vary across regions and under changing external and internal conditions? Do all nodes receive, process and relay messages at the same rate? Questions of this type often arise in relation to many classes of distributed communication networks [13]–[15]. Indeed, the brain must engage in networked computation [16]–[18], a challenge common to multiple types of telecommunication systems [19]. Therefore, it may be possible to learn more about the functional architecture and organizational principles of the brain by treating it as a network of regions that emit units of information.

Here we take the first step in adapting tools from telecommunications research to the problems in neuroscience. Namely, we show how electrophysiological recordings can be plausibly translated into a trace of departing units of information (henceforth referred to as “messages”) and analyzed from the perspective of a telecommunication system. By casting the problem in this light, we may be able to find new ways to describe, quantify and model the flow of information along the distributed brain network. One of the fundamental system statistics for modeling and performance evaluation of communication networks is the distribution of time between successive message departures at each node [13]–[15], [20], [21]. The inter-departure time depends on how messages get processed as well as the nature of their aggregated arrivals to a node and as such it reflects the flux of information through the network. In the present study we devised a method to delineate units of information in gross neurophysiological recordings and to fully characterize the distribution of their inter-departure times.

We first describe an intuitive signal processing approach that can be used to extract such events from the electroencephalogram (EEG). Participants were at rest, with both eyes-open and eyes-closed conditions. The data were resolved in the time-frequency domain using a wavelet transform. We defined message departure times as the local minima in the EEG scalogram, a definition based on the direct physiological interpretation of the EEG. Peaks and bursts in EEG signal power represent the synchronous firing of post-synaptic potentials from a population of neurons. If we take the neuron soma to be grey matter nodes in the network (as the graph model does), then the propagation of post-synaptic potentials to the axon hillock and along the axon may be thought of as the departure of a message. Thus, the troughs preceding each peak mark the point in time at which a unit of information departs from that population of neurons. We show that the distribution of time between successive departures (the inter-departure time) is well described by the family of two-parameter Gamma distributions. These distributions were fitted at each electrode and the two estimated parameters were then treated as dependent variables of neural activity.

If such events do indeed capture some aspect of information flow in brain networks, then we can make several testable predictions. First, the actual paths and sequences of “hops” between nodes will be largely determined by their structural connectivity, so inter-departure time statistics should be region specific and their spatial distribution should be heterogeneous. Second, as external demands change, so too should the manner in which units of information are emitted across the network and the distribution of inter-departure times at individual nodes should also be task-dependent. In particular, we expected the greatest change to be observed at or near occipital channels, given that the biggest difference between the eyes-closed and eyes-open states is the presence of visual input.

## Materials and Methods

### EEG acquisition

The experimental protocol was approved by the Research Ethics Board of the Montreal Neurological Institute and Hospital. Fifty-six (29 male) healthy children 10 years old (mean 10.0, standard deviation 0.393 years) participated in the study (see [22] for details). The participants were asked to keep their eyes open or closed in 8 alternating 30 s epochs (4 each). The electroencephalogram (EEG) was continuously recorded from 128 scalp locations using a HydroCel geodesic sensor net (Electrical Geodesics, Inc., Eugene, OR) referenced to the vertex (Cz). The signal was digitized at a rate of 500 Hz. Impedances did not exceed 60 kΩ. All offline signal processing and artifact correction was performed using the EEGLAB toolbox [23] for MATLAB (Mathworks, Inc.). Data were then average-referenced, digitally filtered [band-pass: 0.5–55 Hz; notch: 60 Hz] and epoched into 30 s segments. Only the middle 20 s of each epoch (5–25 s) were used in the analysis to avoid excessive contamination associated with opening and closing of the eyes. In the absence of a true baseline, the temporal mean was subtracted from each epoch. Ocular (blinks and lateral eye movements) and muscle artifacts were identified and subtracted on a subject-by-subject basis using the Infomax independent components analysis (ICA) algorithm [24] implemented in EEGLAB.

### Wavelet transform

**Figure 1:**
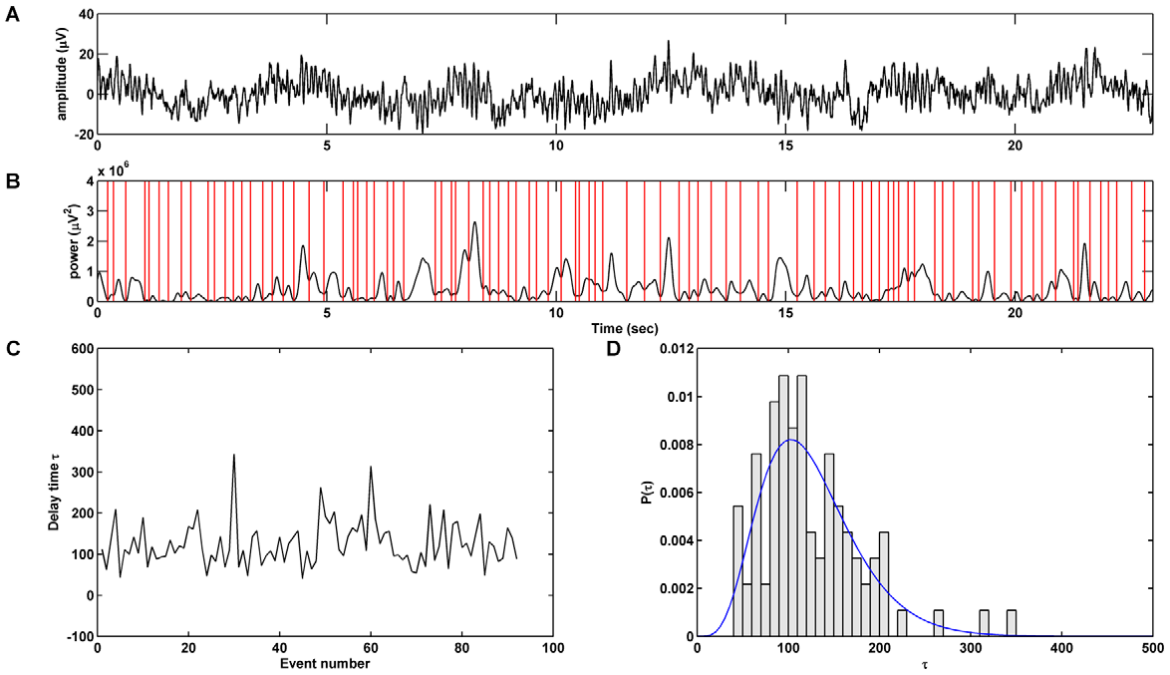
Processing pipeline. The electroencephalogram (**A**) is transformed into the time-frequency domain (**B**) using complex wavelets. Local minima in the scalogram (**B**, red vertical lines) are identified and marked as message departures. The delay 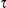 between successive departures is calculated in terms of digitization intervals (**C**). The empirical probability distribution of 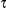 is fitted using the two-parameter Gamma distribution function (**D**).

Dynamic spectral changes were estimated using a wavelet transform [25], implemented in the Wavelet Toolbox for MATLAB (Mathworks, Inc.). Trial epochs were convolved with a complex Morlet wavelet in a sliding window and signal power was estimated as the modulus squared of the real-valued wavelet coefficients (Figure 1B). The Morlet wavelet is a Gaussian-modulated complex sinusoid, so it is considered biologically plausible because it is more sensitive to transients in time series (more so than the windowed Fourier transform) and is widely used as an alternative way to model signals such as the EEG [26]. The mother wavelet had center frequency (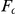) equal to 1 Hz and envelope bandwidth equal to 2 s. Due to Heisenberg's uncertainty principle, there is a trade-off between the temporal precision and the spectral precision of the transform. Because our primary goal was to localize power fluctuations in the time domain, the bandwidth was deliberately chosen to be as narrow as possible to maximize the temporal precision of the transform, while maintaining at least two full cycles. The mother wavelet was compressed and applied at six scales, corresponding to frequencies of 5–30 Hz, in steps of 5 Hz. The corresponding pseudo-frequencies (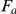) were estimated as the inverse of the product of the scale (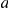) and digitization interval (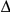): 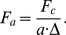
(1)


### Inter-departure time distributions

Departure times were identified by searching for all local minima in the scalogram (Figure 1B). To prevent minute and insignificant troughs from being selected, a local neighborhood threshold was set as a ratio (5%) of the range of the scalogram amplitude. The exact choice of the ratio in the range 2–10% did not impact the functional form or the parameters of the departure time distributions in any significant manner. The time between successive departures (inter-departure time, 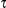) was calculated for each participant, condition, channel and wavelet scale (Figure 1C), producing samples with an average of 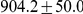 inter-departure times.

Distributions of inter-departure times were then fitted with the two-parameter Gamma probability distribution function using maximum likelihood estimation (Figure 1D). The two free parameters estimated were the shape 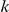 and scale 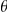. The Gamma probability density has the following form: 
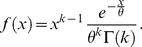
(2)


**Figure 2:**
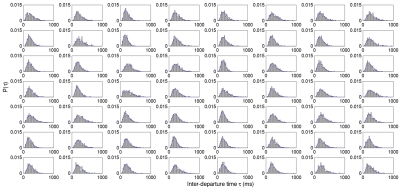
Empirical probability distributions. Empirical probability distributions for the inter-departure time 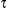 for all subjects at one representative channel (Cz) and one representative frequency (15 Hz). Fitted Gamma density functions are displayed in blue.

**Figure 3:**
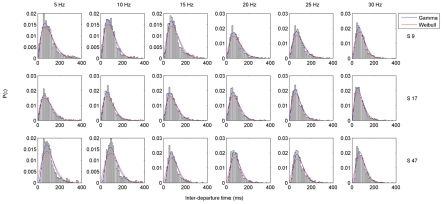
Fits for the Gamma and Weibull distributions. Histograms for the inter-departure time 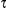 are shown for three subjects (rows) and all frequencies (columns), at one representative channel. Fitted density functions for the Gamma (blue) and Weibull (red) distributions are overlayed.

The Gamma distribution was not selected a priori, but was determined to be the most appropriate distribution when the data were fitted with 30 common distributions and the goodness of fit was assessed by way of the 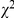 test using EasyFit software (MathWave Technologies). The test statistic was significantly greater than the critical value for all 30 distributions (including the Weibull, Gaussian, generalized Pareto, etc.), indicating significant departure from all those distributions. However, the Gamma distribution had the lowest 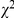 value across all fits and was ranked as the best-fitting distribution. Other common goodness of fit tests, such as the Kolmogorov-Smirnov and Anderson-Darling, were deemed inappropriate because they do not adjust the critical value to account for the degrees of freedom lost when parameters are estimated from the data. Upon visual inspection of the histograms it was clear that the two-parameter Gamma distribution offered an excellent fit to the observed data (Figure 2). The superiority of the Gamma distribution is demonstrated in Figure 3, which shows the fits for the Gamma and the next best-fitting distribution, the Weibull.

### Partial least-squares analysis

We treated each of the two parameters from the fitted Gamma distributions (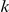 and 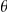) as measures of neural activity. For each parameter we performed separate mean-centered partial least-squares (PLS) [27]–[29] analyses. PLS is a multivariate statistical technique that can be used to relate a design variable (e.g. experimental conditions) to a dependent measure of brain activity (e.g. 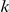 or 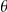) that varies across one or more dimensions (e.g. space and frequency). Singular value decomposition (SVD) is used to compute an optimal least-squares fit to the covariance between those two sets of variables (e.g. 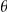 across all electrodes and conditions). Each solution is termed a “latent variable” (LV) and is expressed in terms of a pair of orthogonal vectors of design saliences and electrode saliences (analogous to component loadings in principal components analysis), as well as a scalar singular value (*s*). In the present analysis, each LV represented one contrast between conditions (design salience) in relation to a particular pattern of electrodes and frequencies that expressed that contrast (electrode salience). The “cross-block” covariance between the design block and electrophysiological data block that is captured by an LV is reflected by the singular value. Thus, effect size can be estimated as the ratio of the square of the singular value associated with that particular LV to the sum of all squared singular values derived from the decomposition.

Experimental effects captured by each LV were statistically assessed using resampling techniques. The significance of each statistical effect was determined using permutation tests. Each permuted sample was obtained by random sampling without replacement to reassign the order of conditions within participants (500 replications). The *p*-value was determined by calculating the proportion of permuted singular values that was equal to or exceeded the original singular value. The stability of the multivariate pattern expressed by electrode saliences was indexed by using bootstrap resampling to estimate their standard errors [30]. Bootstrap samples were generated by random sampling with replacement of participants within conditions (500 replications). Saliences were deemed to be reliable if the 99% confidence interval did not include zero. Under the assumption that the bootstrap distribution is unit normal, this condition holds if and only if the absolute value of the ratio of the salience to its bootstrap-estimated standard error is greater than or equal to 2.57 [30].

## Results

### Distributions of 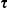


**Figure 4:**
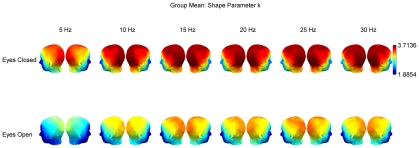
Group means for the maximum likelihood estimates of the Gamma distribution shape parameter 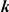. Group means are displayed separately for eyes-closed (top row) and eyes-open (bottom row) conditions, and frequencies from 5 to 30 Hz.

**Figure 5:**
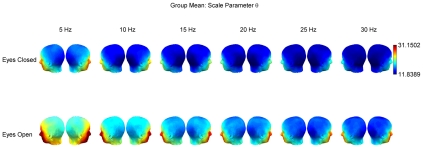
Group means for the maximum likelihood estimates of the Gamma distribution scale parameter 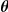. Group means are displayed separately for eyes-closed (top row) and eyes-open (bottom row) conditions, and frequencies from 5 to 30 Hz.

The empirical inter-departure time (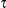) distributions were fitted with the two-parameter Gamma distribution for each condition, subject, electrode and frequency. The Gamma distribution offered a good fit at all frequencies. Despite some individual differences in the parameters of the distribution, the form was remarkably consistent across subjects. This is illustrated in Figure 2, which shows the fits for all 56 subjects at one electrode and one frequency. Nevertheless, there was also substantial variation from subject to subject for both estimated parameters. To illustrate the individual variation of fits across frequencies, we also report the coefficient of variation of each parameter in the Eyes-Open condition, for electrode Cz, for the six frequencies, going from 5 to 30 Hz: 0.22, 0.20, 0.21, 0.20, 0.25 for the shape parameter; 0.33, 0.32, 0.34, 0.34, 0.37 and 0.31 for the scale parameter. The data indicate that both parameters are quite sensitive to individual differences. The spatial distributions of group means for 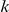 and 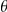 are displayed in Figs. 4 and 5 and discussed in more detail in the following subsection.

Note that since wavelets effectively act as a band-pass filter, the means of 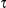 distributions should vary in proportion to frequency, such that departures are expected to occur at a faster rate at higher frequencies, resulting in lower mean inter-departure times. As an example, the group mean inter-departure times for the Eyes-Open condition, channel 60, going from 5 Hz to 30 Hz, were 50.9±1.3, 47.9±1.2, 45.3±1.2, 44.3±1.1, 43.1±1.1 and 40.9±0.8 ms. However, our analyses were concerned with identifying regional and state-dependent statistical effects and did not compare frequencies to each other.

### Shape parameter 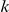


**Figure 6:**
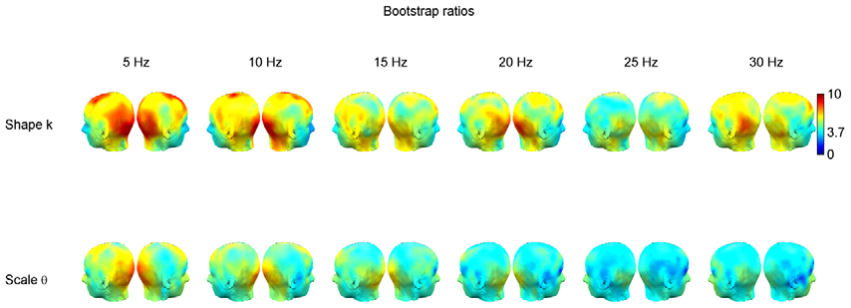
Bootstrap ratio maps for two PLS analyses comparing eyes-closed and eyes-open conditions. Top row: the dependent variable in the first analysis is the shape parameter 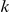 and bootstrap ratios indicate channels at which values are greater in the eyes-closed versus the eyes-open condition. Bottom row: the dependent variable in the second analysis is the scale parameter 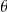 and bootstrap ratios indicate channels at which values are greater in the eyes-open versus the eyes-closed condition.

Across all frequencies, the shape parameter of the fitted Gamma distributions was greater over the posterior (occipital and parietal) channels (Figure 4). Moreover, this measure was sensitive to experimental condition and was greater in the eyes-closed than in the eyes-open condition (Figure 4), an observation statistically supported by the PLS analysis (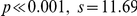). The statistical effect was most reliable across all frequency bands over occipital channels and to a lesser extent over parietal and frontal channels (Figure 6, top row).

### Scale parameter 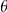


The scale parameter was lower at most posterior and vertical channels and generally much higher over temporal and anterior channels. This pattern was observed at all frequencies (Figure 5). Values were significantly greater in the eyes-open condition (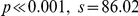) and this effect was most stable over occipital channels (Figure 6, bottom row). There was also some suggestion of frequency dependence in the sense that the bootstrap ratios were slightly higher (i.e. the effect was more robust) at lower frequencies. It is worth noting that the most extreme values of 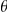 were observed at electrodes close to the eyes (Figure 5), which tend to undergo the heaviest signal processing under most artifact rejection schemes. However, this does not affect the statistical analysis, as the condition differences at these electrodes were not reliable by bootstrap test.

## Discussion

We have described a signal processing method that can be used to identify message departure times from neurophysiological data and quantify the distribution of times between successive departures. The present study demonstrates that the two-parameter Gamma distribution offers a good fit to the inter-departure time distribution. The parameters of inter-departure time distributions were not uniform across the scalp and instead displayed spatial specificity. Namely, distributions recovered from medial posterior electrodes tended to have larger 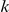 and smaller 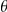 compared to anterior electrodes. This suggests that inter-departure times may be sensitive to regional differences in connectivity and/or processing capacity. In addition, inter-departure times proved to be sensitive to cognitive engagement, with significantly greater 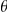 and smaller 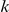 at occipital channels when participants kept their eyes open.

### Variability of inter-departure times

What does systematic variation in 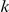 and 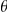 tell us about the functional capacity of the underlying system? For example, what does it actually mean for a cortical region to produce inter-departure times with greater 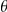 and smaller 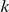 in the eyes-open condition? Here it may be instructive to consider other statistics of the distribution that are easier to interpret. For example, the coefficient of variation (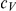, the ratio of the standard deviation to the mean) is a normalized measure of dispersion and for the Gamma distribution is given by 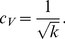
(3)


Thus, inter-departure times were more variable at medial posterior channels compared with the rest of the scalp. Moreover, the distributions became more dispersed in the eyes-open condition and the effect was robust at occipital channels. These results suggest that inter-departure times capture a facet of network traffic. For example, traffic traces in telecommunication networks are found to be more variable under conditions of greater spectrum occupancy [31], [32]. The fact that inter-departure times were more variable at parietal channels is consistent with the notion that structures situated in posterior cortex (particularly close to the midline, such as the precuneus and posterior cingulate) enjoy an exalted status in the connectome. These regions tend to occupy positions along the shortest white-matter paths between all other regions of the brain and participate in the greatest number of structural [8], [33]–[35] and functional subnetworks [8, 11 36, 37].

Given that the biggest difference between eyes-open and eyes-closed is the availability of visual input it is not surprising that condition differences were expressed most reliably over the occipital portion of the scalp. This condition-dependent differentiation may reflect the transient reconfiguration of functional networks in response to changes in external input. For instance, as visual processing becomes more prominent in the eyes-open condition, more information should be routed through the occipital cortices. This should influence the rate of information exchange and total flux through the associated subnetworks, making the underlying biological and cognitive operations less regular and less predictable. This is reflected by our results, which indicate that when the eyes are open, both very short and very long inter-departure times become more likely than when the eyes are closed. The expression of condition differences at multiple frequencies precludes the interpretation that they are the result of a simple difference in power spectral density in the 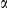 frequency band typically observed in visual tasks. For example, condition differences were not specific to activity resolved at 10 and 15 Hz.

### Importance of the Gamma distribution

**Figure 7:**
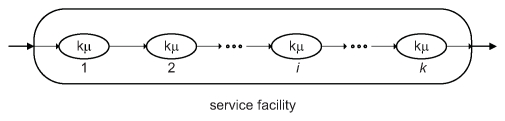
An Erlang-type service facility. The server is comprised of 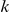 stages/stations arranged in series, each with exponentially distributed service times with rate 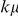. The total time to traverse all stages has a Gamma distribution with shape parameter 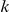 and scale parameter 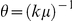. In the context of brain function, the server may represent a population of neurons, while the service stages may represent the sequence of steps required to process a unit of information, such as the diffusion of vesicles at a group synapses.

From the perspective of telecommunication systems, the fact that inter-departure times were best approximated by the Gamma distribution is significant. The Gamma distribution arises naturally and often in such systems, particularly in relation to waiting times. For instance, the round-trip delay time for a packet on the Internet (the time it takes to travel from the source node to the destination and back to the source) is best modeled using the Gamma distribution [38]. In particular, when the shape parameter 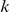 is a positive integer, the Gamma distribution can be thought of as the sum of 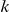 independent exponentially distributed random variables, each with a rate parameter 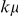. This situation arises when a message must be processed or receive some type of service over a series of stations or stages at a server (termed an Erlang server, Figure 7), each of which has an exponential service time distribution. For instance, the server may represent a population of neurons (as in the graph model). The stages are simply a sequence of processes that take place before a unit of information is emitted. In the context of a neuronal ensemble, these processes may represent the interactions among cells within the ensemble. The time spent at the 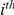 stage, 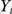, is drawn from the probability density function 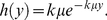
(4)


Since the service times are exponential, the expectation and variance for 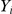 are given by: 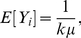
(5)

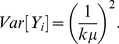
(6)


The total time spent at the server (traversing the 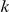 stages) is the sum of 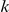 independent identically distributed random variables drawn from the distribution 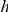. Therefore, the expectation and variance of the total processing time 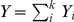 can be calculated by summing across the 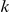 stages: 
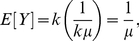
(7)

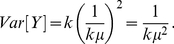
(8)


Importantly, the coefficient of variation of the total service time is given by 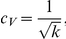
resulting in a hypoexponential service time distribution, named to denote the fact that the coefficient of variation for this distribution is smaller than that of the exponential distribution (i.e. 1) [13]. Hypoexponential service times indicate that the underlying processing stages are arranged in series (Figure 7). If there is any branching and some stations are arranged in parallel, service time distributions will be hyperexponential, with a coefficient of variation greater than 1 (for a detailed derivation see [13]). In the present data, inter-departure times were found to be hypoexponential, which under this theoretical framework is indicative of the former arrangement. This view is biologically plausible, because it suggests that once a unit of information arrives to a node, the sequence of operations performed on that unit is set and does not change from unit to unit. Note however, that although these stages may represent a transformative process, they do not necessarily alter the information content of each unit. Importantly, this derivation should not be misinterpreted as a statement about whether large-scale cognitive processes are coordinated in series or in parallel. Our data merely suggest that there is no variation in the sequence of steps performed on each unit.

The Laplace transform of the exponentially-distributed service time random variable 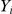 with rate 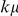 is 
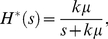
(9)and the transform of the sum of 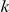 such random variables is the product of their transforms 
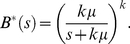
(10)


The transform can then be inverted to give the distribution of total service time: 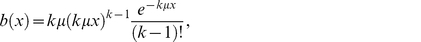
(11)which is a special case of the Gamma distribution (Eq. (2)) where 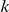 is a positive integer and the scale parameter 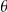 is the inverse of the exponential rate parameter (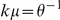).

Overall, this conceptualization of neural dynamics provides a novel narrative of information flow in the brain. This view suggests that units of information may be processed in a series of independent stages. Moreover, the number of processing stages (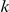) and the service rate at each stage (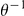) vary across regions of the brain and depend on internal and external conditions. The presence of visual input appears to engender a mode of operation with fewer processing stages but slower service rates. Thus, although it is not the only possible explanation, a telecommunication-based perspective offers a simple and biologically meaningful interpretation for the observed hypoexponential Gamma-distributed inter-trough times and the associated parameters 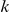 and 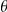.

### A telecommunications view of brain function

The idea to delineate signal units in the EEG and to characterize the sequence of inter-departure times is directly inspired by research in telecommunication networks. However, it is important to consider the physiological validity of the telecommunication model. To what degree are units of information recovered from the EEG scalogram comparable to data transmitted in a typical telecommunication network? In our approach, emitted peaks and troughs are de facto the basic units of information transfer, whereas in neural systems the more likely candidates would be action potential spikes or spike trains [39]. The key is that we would like to know how information emitted across the scalp changes under different experimental conditions. For this context and by virtue of their spatial scale and coverage, gross neurophysiological recordings such as the EEG which represent aggregated postsynaptic potentials from entire populations of neurons are the more appropriate measure of neural activity from which to isolate inter-departure times compared to single cell recordings. It is also interesting to note that, although action potential spikes are often modeled as a Poisson point process, inter-spike intervals (ISIs) measured from single cells often do not appear exponential but take on a functional form rather more similar to the Gamma distribution described here (e.g. Figure 1C in [40]).

The goal of the present study was to establish a foundation upon which the effects of experimental perturbations on communication in the brain could be studied, rather than to advocate any specific structural or functional similarities between telecommunication and brain networks. We sought to delineate physiologically meaningful units of information from gross electrophysiological recordings and to apply analytical tools from telecommunications research to describe how they are emitted across the network. However, some authors have articulated possible parallels between the brain and specific types of telecommunication networks. For example, Graham and Rockmore [41] posited that the brain may actually route and relay information in a manner analogous to packet-switching on the Internet, whereby a message is chopped up into a number of “packets” which are then transmitted along different paths to the destination, where they are re-assembled. The paths taken by individual packets are not pre-determined at the source and instead get adjusted dynamically at each node along the path according to network conditions. Under the current scheme for extracting inter-departure times it is not possible to infer the routes of individual messages. How information flow is directed in the brain and whether the mechanism bears any similarity to a packet-switching network remains to be determined.

However, the benefit of accurately characterizing inter-departure time distributions will be to inform future computational models and to test hypotheses about how information is directed in the brain. By combining physiologically realistic connectivity and realistic inter-departure time statistics, it will be possible to construct simulations with multiple types of routing mechanisms and dynamics unfolding over a cortical foundation. Such models will allow detailed examination of the communication capabilities of the cerebral cortex. For example, they could be used to answer a variety of interesting questions, such as which combinations of nodes and paths are particularly prone to congestion and which nodes become bottlenecks.

### Methodological considerations

How will the present method generalize to other experimental settings, such as an event-related design with multiple shorter trials? One of the keys to fitting distributions to empirical data is sufficient sample size. In other words, to estimate the distribution of packet inter-departure times with a reasonable degree of confidence, one must generate many such packet departures. In a more traditional setting where time series are epoched into shorter segments the same procedure could be applied by calculating 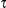 in all individual trials and collating them into a single sample to be fitted. In addition, it remains unclear what impact, if any, time-locked evoked responses would have on 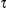 and this certainly warrants further investigation.

The EEG is vulnerable to volume conduction and therefore the spatial precision with which we were able to describe changes in inter-departure time distributions is naturally limited. Moreover, the present method treats all units of information in the same vein, even though peaks in the EEG scalogram vary in their amplitude. In other words, our method implicitly allows the possibility that units of information transmitted in the brain may vary in size. However, even if differences in message size were to be taken into account, this would not change the inter-departure time statistics extracted from the time series.

### Conclusion

In the present study we applied tools from teletraffic engineering to the study of neural activity patterns. We have developed a way to identify electrophysiological events that may be interpreted as departing units of information and we have shown that the times between departures are distributed according to the Gamma probability distribution. In addition, we have demonstrated that this facet of neural activity is meaningful from the perspective of cognitive function. Namely, distributions of inter-event times are highly dependent on cognitive state and spatial location. We conjecture that inter-departure times reflect the flow of network traffic and index the communication capability of the brain's functional architecture.
